# Genetic abnormalities in congenital melanocytic nevi and their associated melanomas

**DOI:** 10.1016/j.jdcr.2024.01.022

**Published:** 2024-02-02

**Authors:** Ashwath J. Sampath, Alexis M. Ruffolo, Jayson Miedema, Paul B. Googe, Nancy E. Thomas

**Affiliations:** a Department of Dermatology, University of North Carolina, Chapel Hill, North Carolina; b Department of Plastic Surgery, Southern Illinois University, Springfield, Illinois

**Keywords:** BRAF, chromosomal rearrangement, congenital melanocytic nevi, melanoma, next-generation sequencing

## Introduction

Congenital melanocytic nevi (CMN) are heterogeneous in their clinical appearance, likelihood of development of melanoma, and their genetics. Only in the last 10 years has the underlying somatic genetics of CMN been elucidated, a direct result of new technologies such as the next-generation sequencing (NGS) of tissues. These technological advances have allowed the opportunity to uncover the molecular heterogeneity of CMN and compare the somatic genetics of CMN with melanomas arising within them. A select number of studies have investigated the somatic genetics of the various types of CMN. These studies concluded that NRAS and BRAF are the most common mutations found in conventional-type CMN, and that HRAS G13R and NRAS are the most common mutations found in the nevus spilus-type CMN.^1^, ^2^, ^3^ Moreover, the BRAF mutation found in the conventional-type CMN is typically a BRAF V600E mutation.^1^ The BRAF traditionally is thought to act as a driver mutation for the formation of melanoma, whereas subsequent gene aberrations such as mutations in the TERT promoter, CDKN2A, and TP53 promote tumor progression. Interestingly, the largest study of the genotype-phenotype correlations of CMN found that no BRAF mutant CMN went on to develop melanoma.^1^^,^^4^ Here, we present 2 unusual cases of CMN, a conventional-type and a nevus spilus-type, that progressed to melanoma and their uncommon mutations.

## Case reports

### Case 1

**Fig 1:**
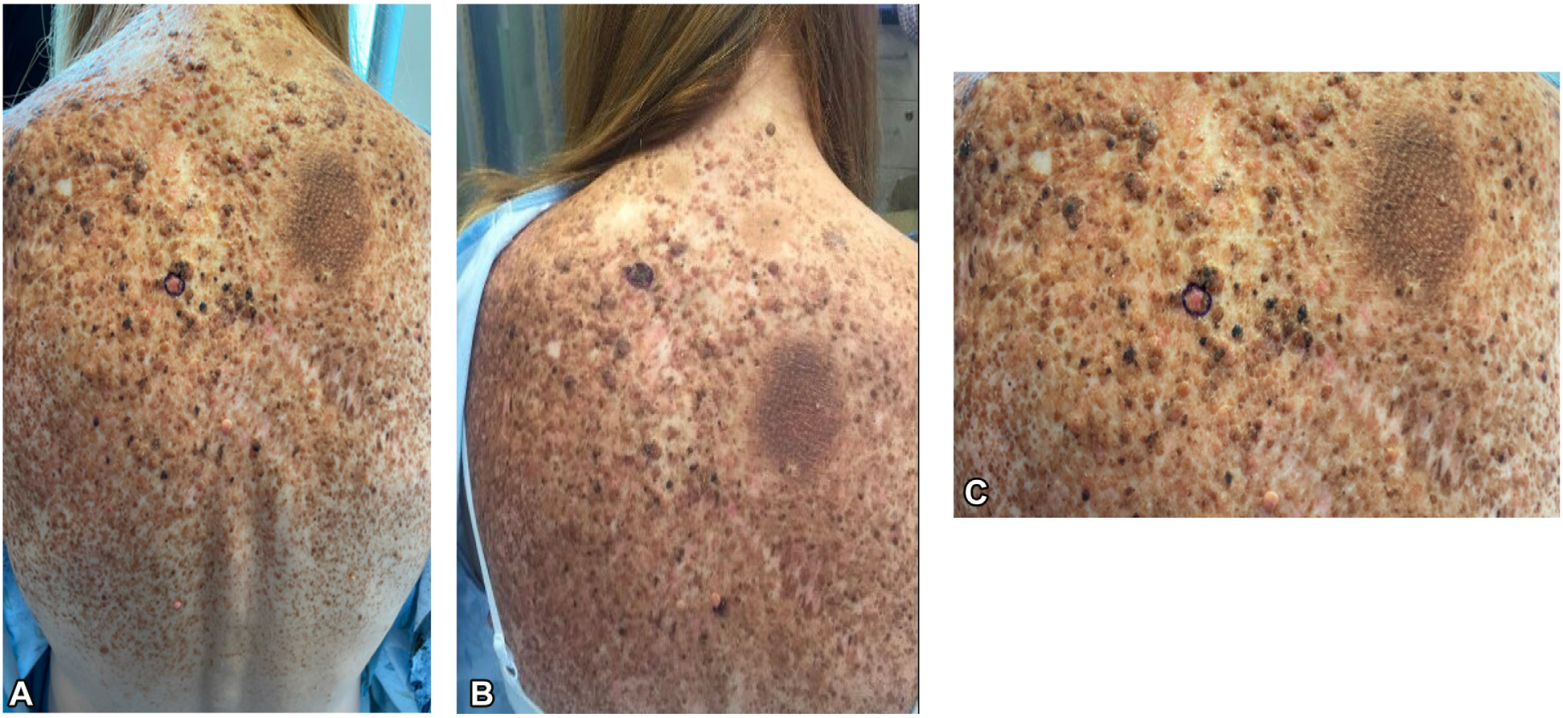
**A,** Large papillated plaque-like lesion with various red, tan-brown, black colors covering much of the patient’s back. **B,** A closer view of patient’s back. **C,** Close up of patient’s right side of the upper back demonstrating the well demarcated circular tan-brown papillated plaque. Centrally there is a pink nodule that was biopsied and consistent with melanoma.

**Fig 2:**
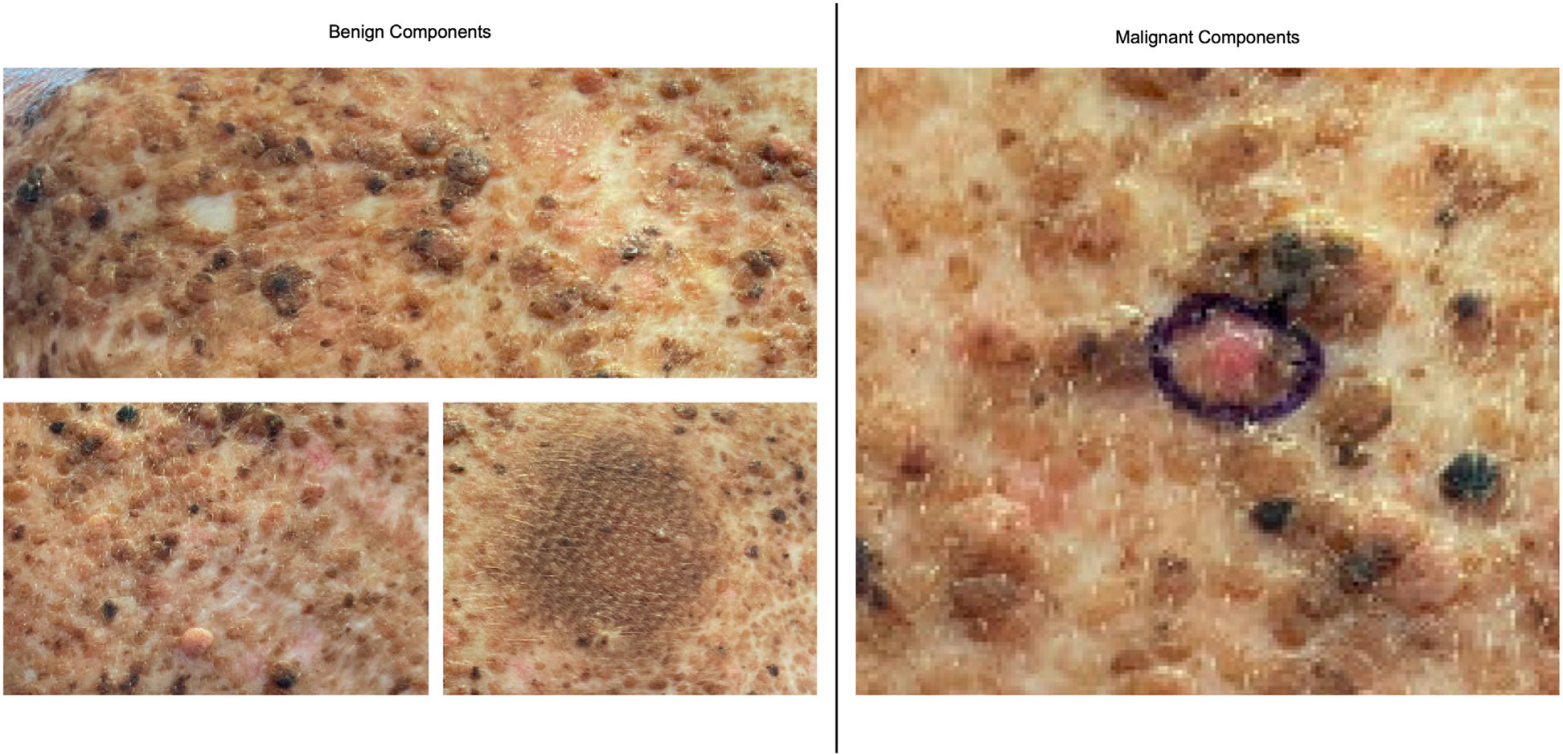
Closer view of patient’s congenital melanocytic nevus demonstrating benign versus malignant components.

A 52-year-old woman with a history of multiple melanomas located on her back and several dysplastic nevi presented to our pigmented lesion clinic for a full body skin check in 2014. She was being treated with interferon-α2b as adjuvant therapy for a stage IIIA melanoma at the time. On examination, a large, atypical papillated and plaque-like lesion with red, brown, and black colors filling much of her back was noted (Figs 1 and 2). History revealed that this lesion was present since birth and grew proportionally with the patient, consistent with a congenital melanocytic nevus. Over the next 10 years, the patient was evaluated regularly at 3-month and 6-month intervals. Numerous biopsies were obtained at these visits. The biopsies demonstrated dysplastic nevi, T1a superficial spreading melanoma (2019), compound nevus (2022), T1a superficial spreading melanoma (2022), T1a superficial spreading melanoma (2023), and melanoma in situ (2023). Immunohistochemistry of her melanoma samples were BRAF V600E negative and PRAME positive. The nevi samples were PRAME negative. The increase in melanomas in 2022 and 2023 was concerning, so tissue sections from the formalin-fixed paraffin-embedded blocks of her background nevus and an invasive melanoma were sent for genetic analysis to gain a more complete understanding of her pathology. The NGS revealed a ZNF777-BRAF chromosomal translocation of the compound nevus biopsied 11/2022. The same ZNF777-BRAF chromosomal translocation, along with a TERT promoter mutation at -124_125CC>TT and MET copy number gain, was appreciated in the nodular melanoma biopsied 10/2013.

### Case 2

**Fig 3:**
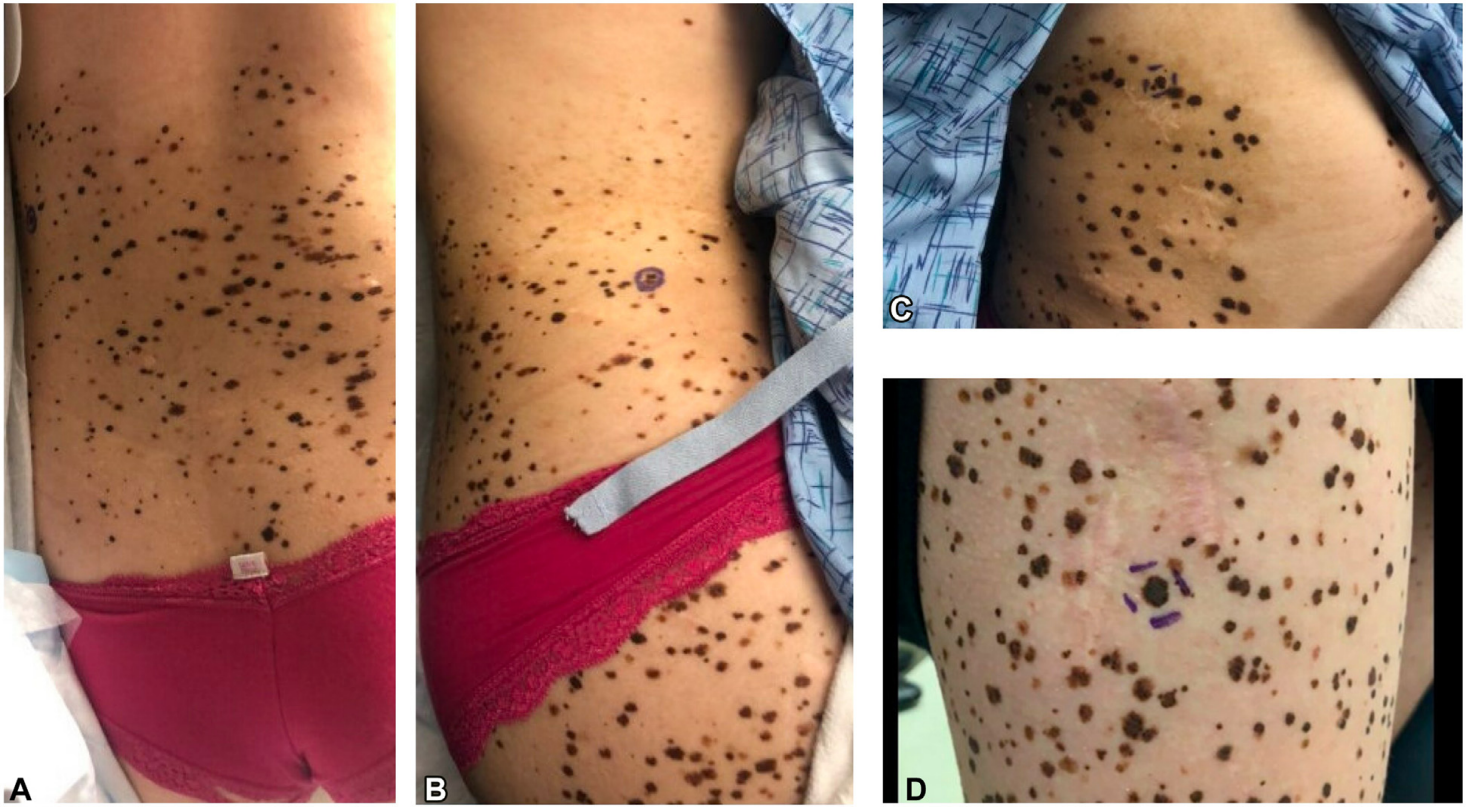
**A, B,** Large café-au-lait with superimposed hyperpigmented macules with on patient’s lower portion of the back and traversing right side of the lower extremity. **C, D,** Close up of patient’s congenital melanocytic nevi demonstrating several scars are seen from previous biopsy sites and surgeries.

A 20-year-old woman with a history of multiple melanomas and several dysplastic nevi presented to our pigmented lesion clinic for a full body skin check. On examination, a large café-au-lait patch with superimposed hyperpigmented macules covering the lower portion of her back and right side of the lower extremity was noted (Fig 3). History revealed that the lesion started out as a café-au-lait at birth, and the hyperpigmented spots appeared later. This is consistent with a nevus spilus-type congenital melanocytic nevus. Over the next 10 years, the patient was seen regularly at 3-month and 6-month intervals. Numerous biopsies were obtained at these visits. The biopsies demonstrated atypical compound nevi, T1a melanoma (2017), and melanoma in situ (2020). Immunohistochemistry of her T1a melanoma (2017) was BRAF V600E negative and PRAME positive. The nevi samples were PRAME negative. To gain a better understanding of the nevus spilus-type congenital melanocytic nevus and the patient’s melanomas, tissue specimens were sent for genetic analysis. The NGS of a compound nevus biopsied 7/2020 revealed a BRAF G469A gain of function mutation. The same BRAF G469A gain of function mutation was seen in the melanoma in situ biopsied 5/2020, along with MAP2K1, ELF3, TRAF3, and TSC2 mutations.

## Discussion

The CMN are relatively common cutaneous lesions that arise from somatic mutations. Here we present 2 cases of CMN, a conventional-type CMN with an underlying ZNF777-BRAF translocation, and a nevus spilus-type CMN with a BRAF G469A gain of function mutation. Both patients had developed multiple melanomas, and a deeper understanding of the CMN was imperative to their future treatment plans.

Patient 1 presented with an exceptional lesion that encompassed much of her back. The history confirmed that the lesion was a CMN. Historically her melanomas presented as pink nodules (Fig 1, *C*) and were clinically aggressive, which is consistent with the TERT promoter mutation and MET copy number gain found in her nodular melanoma. Translocations in CMN are rare, with 4 documented cases in the literature.^3^ We believe her ZNF777-BRAF translocation is the first hit of more than one genetic insult which has resulted in melanoma formation. Subsequent genetic alterations such as in the TERT promoter and MET are the second hits. The presence of the ZNF777-BRAF translocation indicates that, if needed in the future, a MEK inhibitor could be a treatment option because it is downstream from the altered BRAF translocation.

Patient 2 presented with an exceptional nevus spilus-type CMN of the lower portion of her back and right side of the lower extremity. Clinically her melanomas are hard to detect because they have presented as new hyperpigmented macules and patches within her CMN. Her underlying CMN showed a BRAF G469A gain of function mutation. Although frequent in lung cancer, this mutation is incredibly rare in melanoma, and its association with more aggressive disease is uncertain.^5^ Similar to patient 1, we believe her BRAF G469A mutation is the first hit of 2 that results in melanoma formation. The subsequent second hit mutations in MAP2K1, ELF3, TRAF3, or TSC2 lead to development of melanoma. A literature search identified an in-vitro study that showed that BRAF inhibitors have decreased efficacy in melanoma cells with BRAF G469A mutation.^5^ Thus, like patient 1, an MEK inhibitor could be a therapeutic option if needed in the future.

## Conflicts of interest

None disclosed.
